# Oral Administration of PF-01247324, a Subtype-Selective Nav1.8 Blocker, Reverses Cerebellar Deficits in a Mouse Model of Multiple Sclerosis

**DOI:** 10.1371/journal.pone.0119067

**Published:** 2015-03-06

**Authors:** Shannon D. Shields, Richard P. Butt, Sulayman D. Dib-Hajj, Stephen G. Waxman

**Affiliations:** 1 Department of Neurology and Center for Neuroscience and Regeneration Research, Yale University School of Medicine, New Haven, Connecticut, United States of America; 2 Neusentis UK, Pfizer Global R&D, Cambridge, United Kingdom; 3 Rehabilitation Research Center, Veterans’ Affairs Connecticut Healthcare System, West Haven, Connecticut, United States of America; University of Kentucky Medical Center, UNITED STATES

## Abstract

Cerebellar symptoms significantly diminish quality of life in patients with multiple sclerosis (MS). We previously showed that sodium channel Nav1.8, although normally restricted to peripheral somatosensory neurons, is upregulated in the cerebellum in MS, and that Nav1.8 expression is linked to ataxia and MS-like symptoms in mice. Furthermore, intracerebroventricular administration of the Nav1.8 blocker A-803467 temporarily reversed electrophysiological and behavioral manifestations of disease in a mouse MS model; unfortunately A-803467 is not orally bioavailable, diminishing the potential for translation to human patients. In the present study, we assessed the effect of *per os* (p.o.) dosing of a new orally bioavailable Nav1.8-selective blocker, PF-01247324, in transgenic mice expressing Nav1.8 in Purkinje neurons, and in wildtype mice in the experimental autoimmune encephalomyelitis (EAE) model. PF-01247324 was administered by oral gavage at 1000 mg/kg; control groups received an equal volume of vehicle. Behavioral assays of motor coordination, grip strength, and ataxia were performed. We observed significant improvements in motor coordination and cerebellar-like symptoms in mice that received PF-01247324 compared to control littermates that received vehicle. These preclinical proof-of-concept data suggest that PF-01247324, its derivatives, or other Nav1.8-selective blockers merit further study for providing symptomatic therapy for cerebellar dysfunction in MS and related disorders.

## Introduction

Although clinical deficits in multiple sclerosis (MS) are usually attributed to inflammation, demyelination or axonal injury, evidence suggests that additional pathological mechanisms, including molecular mistuning of neurons, contribute to these symptoms [1]. Voltage-gated sodium channels (VGSCs) are specialized protein molecules that support initiation and propagation of electrical impulses in excitable cells such as neurons and muscles. Aberrant expression or activity of VGSCs underlies numerous human disorders, including epilepsy, cardiac arrhythmias, and some pain disorders [2].

It has been shown that one particular tetrodotoxin-resistant (TTX-R) VGSC isoform, designated Nav1.8, is normally expressed within dorsal root ganglion neurons and is normally absent from the cerebellum, but becomes ectopically expressed in cerebellar Purkinje neurons in human patients with MS and in animal models of the disease [3]. When overexpressed in cultured Purkinje neurons in vitro or in mouse models in vivo, Nav1.8 disrupts their function, causing an increase in action potential firing and desynchronizing their normally regular electrical activity [4–6]. At the whole-animal level, this disruption of Purkinje neuron activity is manifest as motor incoordination. Moreover, knockout mice that lack the ability to express Nav1.8 within the cerebellum develop less severe neurological deficits in experimental autoimmune encephalomyelitis (EAE), a model of MS [6]. Together, these data provide strong evidence that Nav1.8 upregulation in the cerebellum in MS and its models represents an acquired channelopathy in this complex disease, likely underlying some of the cerebellar dysfunction associated symptomatically with MS.

On the basis of these findings, we previously delivered an Nav1.8-selective blocker into the cerebrospinal fluid (CSF) of wildtype mice with EAE and demonstrated partial relief of symptoms [6]. This blocker, called A-803467, is not orally bioavailable, thus it was necessary to administer the drug by intracerebroventricular (i.c.v.) injection. We found that i.c.v. delivery of A-803467 could partially reverse the rapid, asynchronous electrical activity displayed by Purkinje neurons in mice with EAE. In addition, we found a modest but significant improvement in symptoms such as wobbly gait and postural abnormalities of the appendages following treatment with A-803467 [6].

The main drawback of this previous approach derives from the pharmaceutical properties of A-803467, which preclude oral administration. Alternatively, a new compound has recently been developed with Nav1.8-selective blocking properties, and is orally bioavailable. This compound, known as PF-01247324 (“Compound A” in [7]), is described fully in [8]. PF-01247324 inhibits the Nav1.8 TTX-R sodium current in human embryonic kidney (HEK293) cells with an IC_50_ of 0.19 μM, and displays >50-fold selectivity for Nav1.8 over Nav1.1, Nav1.5, and Nav1.7. IC_50_’s were also measured for native TTX-R channels in dorsal root ganglion neurons from both mouse and human with values of 0.52 μM and 0.31 μM, respectively [7, 8]. We now report proof-of-concept data demonstrating the efficacy of oral dosing of PF-01247324 to reduce deficits attributable to cerebellar dysfunction in transgenic mice that express Nav1.8 in Purkinje cells, and in mice with EAE.

## Materials and Methods

### Ethics statement

Animal experiments were approved by the Institutional Animal Care and Use Committee of the Veterans’ Affairs Connecticut Healthcare System and conducted in accordance with the NIH Guide for the Care and Use of Laboratory Animals.

Animals were housed 2–4 per cage and maintained on a 12-hour light/dark schedule with free access to food and water. All behavioral testing was performed by an investigator blinded to experimental group.

Experiments were performed on adult L7–1.8TG mice [6] or their wildtype littermates bred in-house (motor coordination studies), or on adult wildtype C57Bl/6 mice purchased from Charles River (EAE studies). EAE was induced according to standard protocols [9]. Mice received a subcutaneous injection of 200 μl of an emulsion of 300 μg rat myelin oligodendrocyte glycoprotein (MOG) 35–55 peptide (Keck Biotechnology Center, Yale University) in incomplete Freund adjuvant (IFA; Sigma-Aldrich) supplemented with 300 μg of *Mycobacterium tuberculosis* H37RA (Difco). The MOG injection, with mycobacterium-supplemented IFA, was repeated in the opposite flank one week later. The mice also received an intraperitoneal injection of 500 ng pertussis toxin (List Biological Labs) in 200 μl 0.1M phosphate-buffered saline immediately after the first immunization with MOG and again 48 h later.

Symptom progression was scored on a 0 to 6 scale [9], with 0.5-point gradations for intermediate scores, as follows: 0, normal; 1, flaccid tail; 2, impaired righting reflex or wobbly gait but no clear weakness; 3, weakness of both hindlimbs or paralysis of a single hindlimb; 4, complete hindlimb paralysis; 5, primarily recumbent and unable to ambulate; 6, death.

Compound dosing and behavioral assessment of EAE mice was performed on day 10 after beginning the EAE induction protocol. At this timepoint, the group average symptom score was approximately 2 (2.04 ± 0.05, n = 39), corresponding to postural abnormalities of the tail, wobbly gait, and impaired righting reflex. We chose this timepoint for compound dosing and behavioral assessment because we postulated that, at this stage of symptom progression, cerebellar dysfunction due to Nav1.8 expression is most prominently manifest. At later stages of the disease, after the onset of weakness and paralysis, the effects of treating cerebellar dysfunction may be less readily apparent by behavioral observation.

PF-01247324 was suspended in 0.5% methylcellulose, 0.1% Tween 80 and administered by oral gavage at a dose of 1000 mg/kg in a volume of 10 ml/kg one hour before behavioral testing. Control groups were administered an equal volume of vehicle.

Behavioral testing was performed as described previously [6]. Immediately after testing, mice were anesthetized with CO_2_ and decapitated, and blood plasma, cerebral cortex, and cerebellum were collected; samples were frozen in liquid nitrogen and stored at -80°C. Drug exposures were determined by standard methods [8]. In animals that received oral gavage of PF-01247324, we found drug concentration at the target tissue (in this case cerebellum) to be at or above 0.5 μM in all subjects (>the IC_50_ as measured by in vitro electrophysiology), thus no animals were excluded from the study for insufficient exposure.

## Results

The pharmacokinetic properties of PF-01247324 upon oral dosing in C57Bl/6 mice are described in Table 1. We chose to use a single, high dose of this compound to determine whether it would be possible in principle to target Nav1.8 channels in the cerebellum by oral administration of an Nav1.8 blocker, for the reversal of motor coordination deficits caused by the aberrant expression of Nav1.8 in Purkinje neurons. Analgesic efficacy of this dose of PF-01247324 has been previously reported in the carrageenan model of inflammatory pain and in the sciatic nerve ligation model of neuropathic pain in rats [8].

**Table 1:** Pharmacokinetic properties of PF-01247324 dosed in C57BL6 mice (n = 3 per dose level).

Dose (mg/kg)	Route	Plasma unbound C_max_ (nM)	Brain unbound C_max_ (nM)	Brain:Plasma ratio	T_max_ (h)	Plasma Protein Binding	IC_50_ at rTTX-R in vitro (nM)
30	p.o.	95	29	0.28	0.5	96.5%	520
100	p.o.	243	87	0.36	0.5		
300	p.o.	523	136	0.26	0.5		

**Fig 1:**
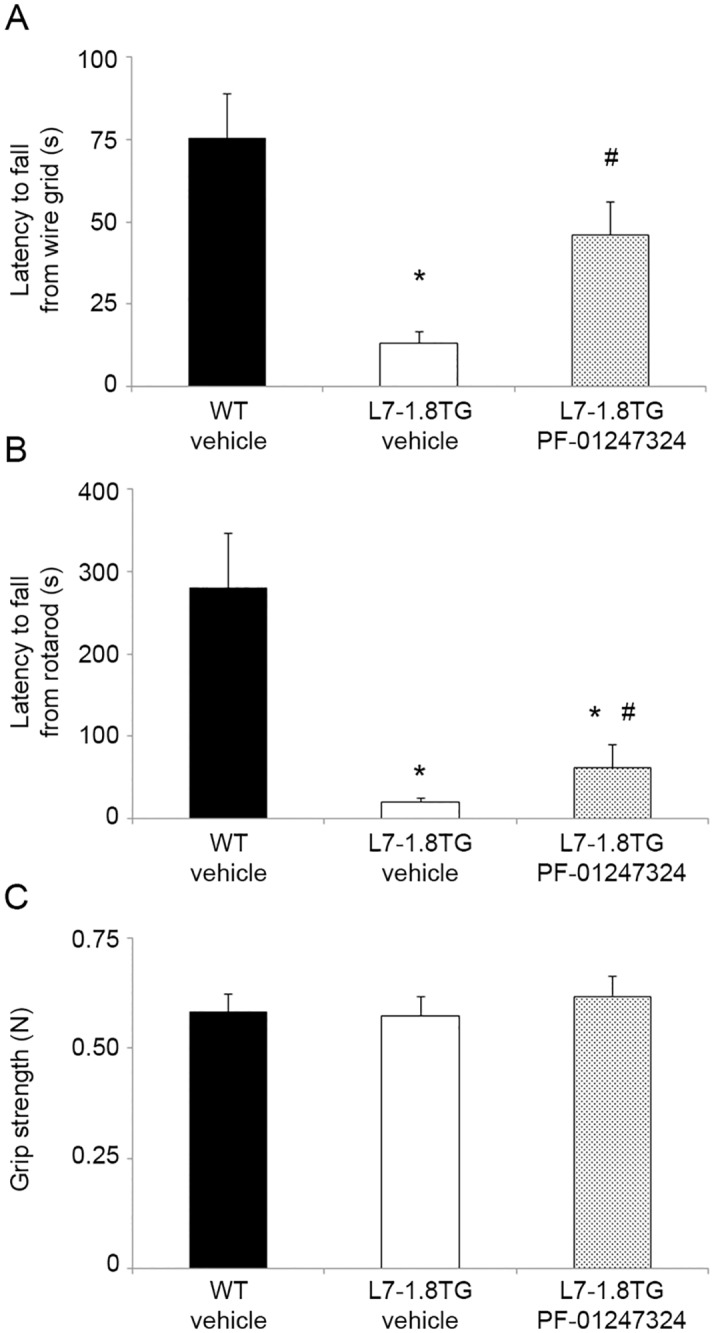
Oral administration of PF-01247324 partially reverses motor incoordination in transgenic mice overexpressing Nav1.8 in cerebellar Purkinje neurons. **A**. Latency to fall from an inverted wire grid was measured in L7–1.8TG mice administered either PF-01247324 or vehicle, and in their wildtype littermates. Behavioral testing was performed one hour after dosing. Differences between the three groups were significant (one-way ANOVA, p = 0.0014). L7–1.8TG mice in the vehicle group performed significantly worse than WT mice. L7–1.8TG mice that received oral dosing of PF-01247324 had significantly improved performance in this assay. **B**. In the rotarod test, significant differences in motor coordination were observed (one-way ANOVA, p = 0.0006). L7–1.8TG mice in the vehicle group were able to stay on the rotating beam for less time than WT mice, indicating a deficit in motor coordination. Oral administration of PF-01247324 to L7–1.8TG mice significantly improved their performance compared to vehicle-treated mice of the same genotype. **C**. Grip strength was similar in each of the three groups. One-way ANOVA, p = 0.7560. *, p<0.05 compared to WT vehicle group; #, p<0.05 compared to L7–1.8TG vehicle group, Dunnett’s post-hoc test. WT+vehicle, n = 8; L7–1.8TG+vehicle, n = 7; L7–1.8TG+PF-01247324, n = 6.

Transgenic mice overexpressing Nav1.8 in cerebellar Purkinje neurons in the absence of any other MS-like symptoms were used. These mice are designated L7–1.8TG mice and were described previously [6]. L7–1.8TG mice are significantly impaired in performing coordinated motor behaviors, including difficulty navigating an inverted wire grid and shorter latency to fall while walking on a rotating beam (rotarod). In blinded experiments, we administered either PF-01247324 or vehicle to L7–1.8TG mice via oral gavage and performed behavioral assessment of motor coordination one hour later. An additional control group consisted of wildtype littermates of L7–1.8TG mice (WT) that were administered vehicle. Fig. 1 shows the results. In the inverted wire grid test, L7–1.8TG mice that received vehicle performed significantly worse than WT mice that received vehicle, reproducing our previous results on the deleterious effects of ectopic expression of Nav1.8 in the cerebellum (Fig. 1A). However, L7–1.8TG mice that were administered PF-01247324 showed a significant improvement in motor coordination in this assay compared to vehicle-treated L7–1.8TG mice (one-way ANOVA, p<0.05; followed by Dunnett’s post-hoc test). Similarly, on the rotarod test, vehicle-treated L7–1.8TG mice performed much worse than vehicle-treated WT mice, and administration of PF-01247324 significantly improved the ability of L7–1.8TG mice to sustain control while walking on the rotating beam (Fig. 1B). The effect of the transgenic mutation, as well as the effect of PF-01247324, was independent of any effect on the subjects’ grip strength (Fig. 1C). Because in L7–1.8TG mice the underlying cause of motor incoordination is ectopic Nav1.8 expression in cerebellar Purkinje neurons, and administration of a selective Nav1.8 blocker improved coordination, we suggest that these data may represent behavioral evidence of target engagement by the compound.

**Fig 2:**
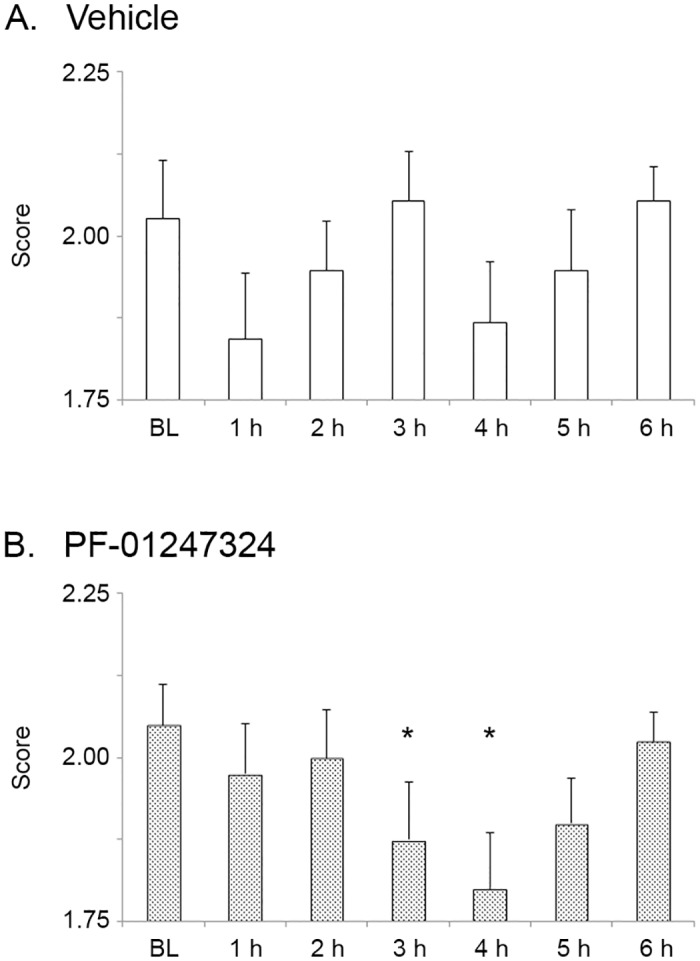
Oral administration of PF-01247324 slightly improves MS-like deficits in the EAE model. EAE symptom progression was scored on a 0 to 6 scale [9], with 0.5-point gradations for intermediate scores, as follows: 0, normal; 1, flaccid tail; 2, impaired righting reflex or wobbly gait but no clear weakness; 3, weakness of both hindlimbs or paralysis of a single hindlimb; 4, complete hindlimb paralysis; 5, primarily recumbent and unable to ambulate; 6, death. **A**. C57Bl/6 mice with EAE that were administered vehicle had stable symptom scores over the 6 h observation period. (All timepoints p>0.05 compared to baseline (BL), paired t-tests. n = 19.) **B**. C57Bl/6 mice with EAE that were administered PF-01247324 showed a slight improvement in symptom scores after dosing, when analyzed with paired t-tests to compare individual timepoints to pre-drug BL. (1 h, p = 0.3299; 2 h, p = 0.5426; 3 h, p = 0.0493; 4 h, p = 0.0084; 5 h, p = 0.0555; 6 h, p = 0.6649; paired t-tests compared to BL. n = 20.) However, a two-way ANOVA did not show significant improvement compared to vehicle control. *, p<0.05 compared to BL, paired t-test.

Although L7–1.8TG mice can be seen as a simple model for one of the manifestations of MS, the EAE model is more realistic because it recapitulates more features of this complex disease, including immune cell invasion of the central nervous system, demyelination, and Nav1.8 upregulation in Purkinje neurons. Therefore, in a second series, we studied wildtype C57Bl/6 mice with EAE [9] and tested oral dosing of PF-01247324 for possible efficacy to improve cerebellar symptoms in this more complex, disease-relevant model. The results of this series of experiments are shown in Fig. 2. In C57Bl/6 mice with EAE that were administered vehicle only, symptom manifestation remained fairly stable over the six-hour observation period following dosing (Fig. 2A). However, in C57Bl/6 mice with EAE that received PF-01247324 by the oral route, a modest but significant improvement of cerebellar symptoms (ataxia, righting reflex, tail posture) was detected at 3 and 4 hours after administration of the drug when analyzed by performing paired t-tests comparing the hourly timepoints to pre-drug baseline (Fig. 2B). However, a two-way ANOVA with repeated measures showed only a marginally significant effect of Time (p = 0.05), but no Time*Treatment interaction (p = 0.19). We conclude that PF-01247324 showed a trend toward slightly improved symptoms in the EAE model, but the effect size was small and not statistically distinguishable from vehicle.

Together these data indicate that oral dosing of PF-01247324 ameliorates cerebellar-like deficits in a mouse model in which Nav1.8 is overexpressed in Purkinje neurons, and shows a trend toward improving cerebellar-like deficits in EAE. These proof-of-concept data thus support our hypothesis that the use of Nav1.8 blockers may provide symptomatic relief for cerebellar dysfunction in MS and related disorders.

## Discussion

MS affects more than 2 million people worldwide. Symptoms of MS vary greatly from patient to patient, and at different times within the same patient. Nevertheless, up to 80% of MS patients experience cerebellar symptoms such as tremor, nystagmus, or ataxia at some point during their disease [10–11]. Importantly, cerebellar symptoms of MS severely impact quality of life for patients, often limiting their ability to drive a car, walk unassisted, or perform tasks associated with their employment. Thus far, effective treatments have been quite limited. The only FDA-approved symptomatic treatment for improving walking in people with MS, 4-aminopyridine, is effective in only a subpopulation of patients and is a potassium channel blocker designed to reverse conduction failure in demyelinated axons. Thus, treatment of cerebellar symptoms in multiple sclerosis represents a sizeable unmet medical need.

Pharmacotherapy of MS with non-selective VGSC blockers (antiepileptics) has previously been attempted with limited success. Carbamazepine is reported to be effective against paroxysmal symptoms of MS in some patients, but leads to adverse neurological effects mimicking relapse in many others [12–14]. Lamotrigine was found to be ineffective as a neuroprotective agent in MS and to produce adverse effects such as dose-related deterioration of gait and balance in the majority of patients in a double-blind, placebo-controlled clinical trial [15]. VGSCs are thought to redistribute along the denuded portions of demyelinated axons [16–18], supporting conduction of action potentials despite the absence of myelin [19]. The mechanism by which non-selective VGSC blockers worsen MS symptoms may involve counteracting this compensatory mechanism, or may reflect blockage of VGSCs at normal nodes of Ranvier upstream to demyelinated regions, an effect that would tend to reduce the safety factor for action potential invasion of demyelinated axonal regions [20].

The advent of isoform-selective blockers of VGSCs presents the possibility of selectively blocking Nav1.8 while leaving other sodium channel subtypes unaffected. PF-01247324 has been demonstrated to block both native human and rodent TTX-R channels [8]. Human recombinant sodium channel isoforms were evaluated using the whole-cell patch-clamp technique, and the data show that PF-01247324 is >50-fold selective for Nav1.8 over Nav1.1, Nav1.5, and Nav1.7. PF-01247324 demonstrates drug-like pharmacokinetic properties that are amenable for preclinical studies and is predicted to be suitable for clinical studies. The data show that PF-01247324 is 91% bioavailable in the rat following oral dosing, with a T_max_ of approximately 0.5 hours and a low clearance with primarily hepatic metabolic pathways. These preclinical observations result in a rat half-life of 4 hours and a predicted human half-life of ≥10 hours. In preclinical studies in multiple rat nociceptive models the effective concentrations of PF-01247324 observed were between 0.12 and 0.89 μM, representing a range from ~0.25- to 2-fold the rat in vitro TTX-R IC_50_ value and consistent with the effective dose range presented here. Furthermore, in the mouse, 1000 mg/kg of PF-01247324 dosed orally resulted in unbound concentrations of 1.22 μM, which did not impair rotarod behavior, indicating that the dose of PF-01247324 used here does not cause global impairment of neurological functions.

## Conclusions

Nav1.8 in the cerebellum appears to contribute to motor incoordination in animal models of MS, while the VGSCs responsible for conduction along myelinated and demyelinated axons in most CNS neurons include Nav1.2 and Nav1.6 [18]. PF-01247324 represents a new, pharmacologically selective, drug-like compound for evaluation in the in vivo mouse models described in this study. The amelioration of the EAE symptoms observed in our current study after treatment with PF-01247324 are encouraging, with potential for clinical translation. Selective therapeutic blockade of Nav1.8 without significant inhibition of other isoforms might provide a basis for the successful treatment of cerebellar dysfunction without inciting adverse effects such as those seen with the use of traditional, non-selective VGSC blockers.
